# Results of a Human Papillomavirus Self-Collection Educational Intervention for Health Care Providers in Appalachia

**DOI:** 10.1089/whr.2024.0121

**Published:** 2025-01-24

**Authors:** Mira L. Katz, Abigail Shoben, Amie M. Ashcraft, Emma Mitchell, Mark Dignan, Sarah Cooper, Mark Cromo, Jean Walunis, Deborah Flinner, Dannell Boatman, Lindsay Hauser, Mack T. Ruffin, Paul L. Reiter

**Affiliations:** ^1^ College of Public Health and the Comprehensive Cancer Center, The Ohio State University, Columbus, Ohio, USA.; ^2^ College of Public Health, The Ohio State University, Columbus, Ohio, USA.; ^3^ School of Medicine, West Virginia University, Morgantown, West Virginia, USA.; ^4^ School of Nursing, University of Virgnia, Charlottesville, Virginia, USA.; ^5^ College of Medicine, University of Kentucky, Lexington, Kentucky, USA.; ^6^ Comprehensive Cancer Center, The Ohio State University, Columbus, Ohio, USA.; ^7^ Cancer Center, University of Virginia, Charlottesville, Virginia, USA.; ^8^ Family and Community Medicine, Penn State Health, Hershey, Pennsylvania, USA.

**Keywords:** Appalachian region, cervical cancer, cancer screening, human papillomavirus, education, health personnel

## Abstract

**Objective:**

There is an increasing interest in human papillomavirus (HPV) self-collection as a strategy for women not up-to-date with cervical cancer screening. We report the findings of an HPV self-collection educational intervention for health care providers and staff.

**Materials and Methods:**

As part of the Health Outcomes through Motivation and Education (*HOME*) *Initiative*, health care providers from 10 health care systems in Appalachian regions of four states attended online sessions during 2021–2023. Participants (*n* = 167) completed pre- and postintervention surveys focused on knowledge and attitudes about HPV self-collection and cervical cancer screening. The postintervention survey also addressed satisfaction with the educational intervention.

**Results:**

Participants correctly answered an average of 4.6 out of 7 knowledge items on preintervention surveys and an average of 6.0 items on postintervention surveys (*p* < 0.001). Attitudes were more positive on postintervention surveys and included that participants reported that they were better informed about HPV self-collection and more confident they could talk to patients about HPV self-collection (both *p* < 0.05). Nearly all (>97%) participants reported being satisfied with the educational intervention and being pleased their health center was included in the *HOME Initiative*.

**Conclusions:**

An online educational intervention for health care providers and staff about HPV self-collection as a cervical cancer screening strategy was efficacious in improving knowledge and attitudes and was well-received by participants. Given its online delivery and that it can be completed individually or in a group setting, this educational intervention with minor adaptations has potential for wide dissemination to educate health care providers and staff about HPV self-collection.

## Introduction

The Appalachian region of the United States includes 423 counties in 13 states from the northern portion of Mississippi to the southern part of New York.^1^ Over 26 million people live in the Appalachian region and residents experience several cancer-related disparities.^2^ One of the long-standing disparities among women living in Appalachia is the higher cervical cancer incidence and mortality rates compared with national rates for the United States.^2^ The reasons for the higher cervical cancer rates among women living in this geographic region are multifactorial and include less access to health care, health care professional shortages, and several social determinants of health and behavioral factors that have an influence at different levels of the socioecological model of health.^1–4^

The United States Preventive Services Task Force (USPSTF) recommendations for cervical cancer screening at the time of the study included cervical cytology alone every 3 years for women aged 21–29 years; and cervical cytology alone every 3 years, high-risk human papillomavirus (hrHPV) testing alone every 5 years, or hrHPV testing in combination with cytology (cotesting) every 5 years among women aged 30–65 years.^5^ The current recommendation for HPV testing involves samples that are collected by a health care professional in a health care setting. Recently, the U.S. Food and Drug Administration approved HPV self-collection by women in a health care setting.^6^ However, a potential alternative approach that is being evaluated in research studies is HPV self-collection that involves patients collecting their own sample at home and sending it to a laboratory for testing. Although HPV self-collection outside of a health care setting is not an approved or recommended screening approach in the United States, research has demonstrated that women mailed an HPV self-collection device can successfully complete a test at home by themselves in privacy and mail the device to the laboratory for testing.^7–9^

Studies have documented that self-collected samples by women have similar performance (*e.g.,* sensitivity, specificity) compared with clinician-collected samples^10–12^ and women have provided positive feedback about the HPV self-collection process.^10,13–16^ Due to the known psychological and logistical barriers that have led to lower cervical cancer screening rates among women living in Appalachia,^4,13,17^ there was an interest in exploring HPV self-collection as a potential strategy to reach under-screened women in this geographic region.

At the health care provider level, qualitative studies have reported both positive and negative opinions about using HPV self-collection as a cervical cancer screening strategy.^13,18–21^ Positive points raised include having another screening strategy for women who previously declined traditional cervical screening, and using HPV self-collection has the potential to bring women back into the health care system for additional health-related issues.^13,18–21^ Concerns expressed by providers were focused on the potential for inaccurate sample collection, not having time to answer patient questions, lack of the opportunity to examine the patient for other health-related issues, information sent to a patient’s home potentially being misunderstood by others especially in the case of an unfriendly home environment, financial-related issues, and the need for provider education.^13,18–21^

Given that many women in Appalachia are not up-to-date with cervical cancer screening,^4,17^ we developed a multilevel intervention titled the Health Outcomes through Motivation and Education (*HOME*) *Initiative*. The initiative is centered on a mail-based HPV self-collection program for women who are not up-to-date with cervical cancer screening. However, prior to starting the mail-based program, we thought it was necessary to educate health care providers and staff in participating health care centers about HPV self-collection. This report describes the findings of an educational intervention for health care providers and staff about HPV self-collection. As only one previous educational intervention about HPV self-collection for health care providers has been published,^22^ the results of the current study will be important in guiding future efforts regarding HPV self-collection programs.

## Materials and Methods

### Setting

The *HOME Initiative* was one of the three initiatives included in a larger research study titled *Take CARE* (Clinical Avenues to Reach Health Equity) to address the higher cervical cancer burden among women living in Appalachia. The *Take CARE* study partnered with 10 health systems that included 39 health centers in the Appalachian regions of four states (Ohio, Kentucky, Virginia, and West Virginia). Most (81%) participating health centers were located in counties with Rural-Urban Continuum Codes 4–9.^23^ In addition, the health centers were located in 13 counties classified as distressed (worst economically depressed 10% of the U.S. counties), six classified as at-risk counties (rank between the worst 10% and 25% of the U.S. counties), and seven classified as transitional counties (rank between the worst 25% and the best 25% of the U.S. counties) as indicated by the Appalachian Regional Commission.^24^

The *HOME Initiative* focused on increasing cervical cancer screening using a mail-based HPV self-collection program, and one component of the initiative was an educational intervention for health care providers and staff about HPV self-collection, which is the focus of this report.

### Participants

Participants were health care providers and staff from the participating health care systems. We coordinated the educational sessions with the health systems to maximize participation. Originally, the provider intervention was planned to be delivered at in-person meetings since academic detailing has been shown to improve rates of cancer screening.^25–28^ However, due to the COVID-19 pandemic, delivery of the educational sessions was changed to an online format that could be viewed by participants individually or as a group. Most participants viewed the intervention individually, with about one-fourth of participants completing the intervention in a group setting.

A total of 167 participants completed an educational session between 2021 and 2023. Participants completed an online consent form and were assigned a subject identification number, before completing an online preintervention survey. Participants then viewed the intervention content (described below) and completed an online postintervention survey accessed by an individualized link by subject identification number. All participants completed the preintervention survey and 136 (81%) completed the postintervention survey. For participants who completed the educational session in a group setting, each filled out surveys on their own computer or mobile device. The Institutional Review Board (IRB) at The Ohio State University was the IRB of record and approved this study.

### Educational intervention

**FIG. 1.**
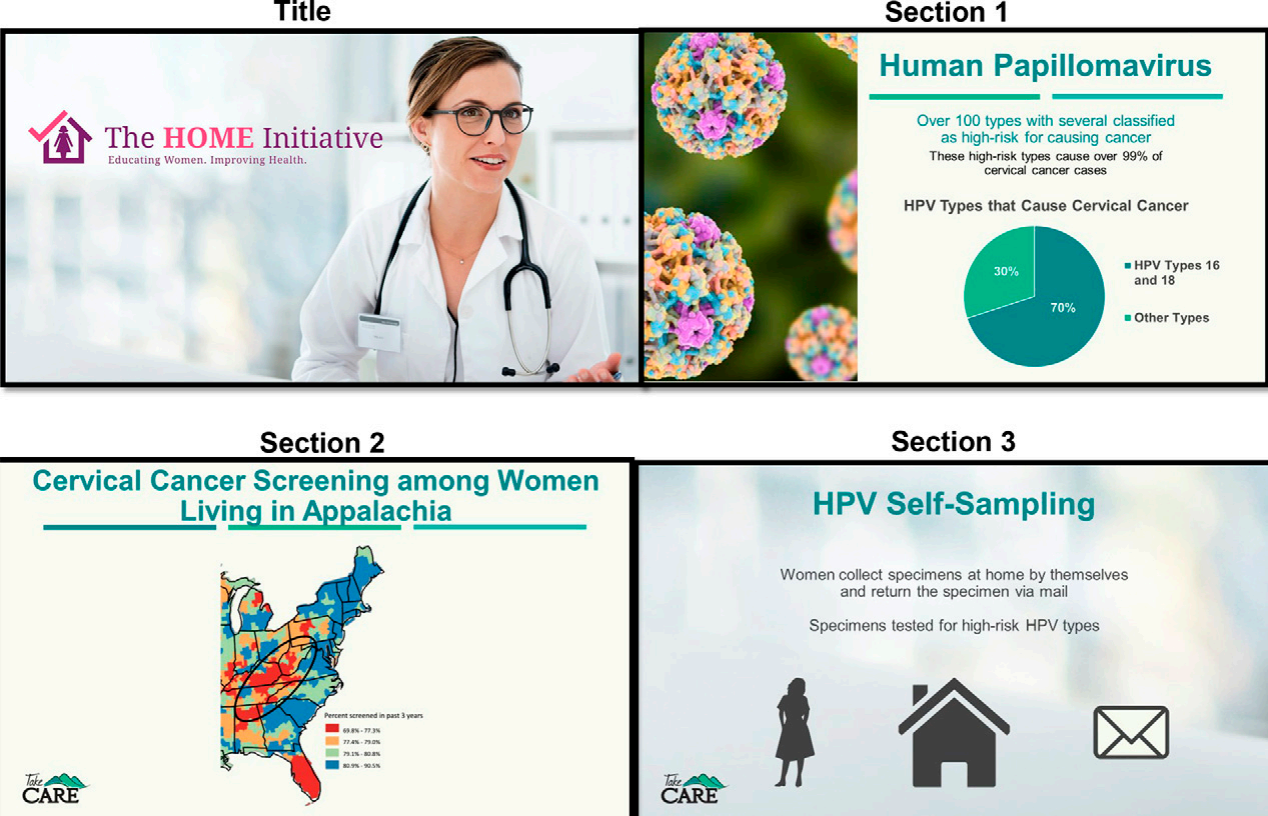
Screenshots from the *HOME Initiative* provider educational video.

We modified a health care provider intervention about HPV self-collection that was developed for a previous pilot study.^22,29^ The intervention content was delivered *via* an educational video with professional narration. We collaborated with a professional media company in an iterative process to produce the video, which lasted 11 minutes and 54 seconds. The video provided information in three distinct sections: (1) an overview of cervical cancer and HPV infection in the United States; (2) a review of current cervical cancer screening guidelines; and (3) an overview of the *HOME Initiative* and HPV self-collection (Fig. 1).

The first section of the video provided general information about cervical cancer incidence and mortality rates in the United States with a special emphasis on the higher rates among women living in the Appalachian region of the United States. Information about HPV infection included that it is a common infection among sexually active individuals, that individuals with an HPV infection may be asymptomatic, that most HPV infections clear without treatment, that HPV infection has been documented in all age groups, and that there are different HPV types that can cause cervical cancer.^3,30,31^ The second section of the video focused on the cervical cancer screening guidelines that were updated in 2018 by the USPSTF.^5^ The review included the appropriate ages for screening, screening test options, and the frequency for when each screening strategy should be completed for a woman to be considered up-to-date with cervical cancer screening. This section also emphasized that cervical cancer screening rates are lower among women living in the Appalachian region compared with national rates for the United States.^32,33^

The final section of the video focused on HPV self-collection and the *HOME Initiative*, including an overview of the self-collection process, the sensitivity and specificity of self-samples, and summarized past experiences with HPV self-collection that women reported from previous studies.^10–13^ The video noted the HPV testing included in the current cervical cancer screening guidelines in the United States includes only samples collected by a health care provider in a health care setting. Additional content about the *HOME Initiative* included that females mailed a HPV self-collection device in the subsequent mail-based program would be patients in the participating health centers, the information that would be provided in the test results for HPV self-collected samples, that test results would be sent to the health care centers, tips on how health care providers can communicate with patients about HPV self-collected test results, and the importance of follow-up care for abnormal test results.

### Measures

The preintervention survey collected information about participants’ demographic characteristics (Table 1). The pre- and postintervention surveys focused on the information provided in the intervention content and addressed knowledge and attitudes about cervical cancer, cervical cancer screening, HPV, and HPV self-collection. The pre- and postintervention surveys included identical knowledge and attitude items. There were seven items that measured knowledge and each item was determined to be correct or incorrect, as indicated in Table 2. Four of the seven items had response options of “true,” “false,” or “do not know,” with responses of “do not know” coded as incorrect. The remaining three items were in a multiple-choice format. We examined each knowledge item separately, as well as an overall knowledge score that summed participants’ total number of correct responses (possible range of 0 − 7).

**Table 1.** Characteristics of Health Care Providers and Staff (*n* = 167)*

Mean age in years (SD)	42 (12.0)
Mean years worked at health center (SD)	
Physicians	6.1 (5.3)
Nonphysicians	4.8 (5.3)

	*n* (%)
Gender	
Female	154 (92)
Male	13 (8)
Race/ethnicity	
Non-Hispanic white	155 (95)
Other	9 (5)
Marital status	
Married/living together	122 (74)
Separated	21 (13)
Single/windowed	21 (13)
Occupation	
Physician	25 (15)
Physician assistant	5 (3)
Nurse practitioner	59 (36)
Registered nurse/licensed practical nurse	55 (33)
Other staff	22 (13)
Education (nonphysicians)	
High school	12 (9)
College graduate	59 (42)
Graduate/professional school	69 (49)
Employment status	
Full-time	157 (96)
Part-time/Other	7 (4)

^*^ Numbers may not sum to total due to missing data.

SD, standard deviation.

**Table 2.** Comparison of Knowledge Items from Preintervention and Postintervention Surveys

		Correct response *n* (%)		
	Correct answer	Preintervention (*n* = 167)	Postintervention (*n* = 136)	*p* value
Many counties in Appalachia have higher incidence rates of cervical cancer compared with the rest of the country.	True	138 (83)	135 (99)	0.005
Women who have an HPV infection always have symptoms.	False	161 (96)	131 (96)	0.97
HPV self-sampling would allow women to complete a test at home by themselves and return it through the mail.	True	143 (86)	135 (99)	0.007
HPV self-sampling is a recommended cervical cancer screening strategy in the United States.	False	41 (25)	92 (68)	<0.001
What percent of cervical cancer cases do you think is caused by infection with human papillomavirus (HPV)? (Response options: 23%/52%/78%/99%/ I don’t know)	99%	33 (20)	79 (58)	<0.001
According to current guidelines from the United States Preventive Services Task Force, at what age should women start being screened for cervical cancer? (Response options: 16/18/21/25 years old/I don’t know)	21 years old	139 (84)	126 (93)	0.004
Imagine a patient is a 35-year-old female with no abnormal medical history. For this patient, what is a recommended cervical cancer screening approach according to the US Preventive Services Task Force? (Response options: Pap test every year/ HPV test every year/HPV test every 3 years/Cotesting with a Pap test and HPV test every 5 years)	Cotesting with a Pap test and HPV test every 5 years	106 (63)	119 (88)	<0.001
Total score: Mean (SD)		4.6 (1.3)	6.0 (1.1)	<0.001

HPV, human papillomavirus; U.S., United States; Pap, Papanicolaou; SD, standard deviation.

Four items measured attitudes about cervical cancer screening and HPV self-collection (Table 3). Three of these items used a 5-point scale with responses of strongly agree, agree, not sure, disagree, and strongly disagree. We dichotomized responses into “agree” (strongly agree or agree) or “disagree” (not sure, disagree, and strongly disagree). One of the attitude items examined participants’ perceptions of how much of a priority cervical cancer screening was for their health center using a scale from 1 (lowest priority) to 10 (highest priority).

**Table 3.** Comparison of Participants’ Attitudes About HPV Self-Collection from Preintervention and Postintervention Surveys

	Strongly agree / agree *n* (%)		
	Preintervention (*n* = 167)	Postintervention (*n* = 136)	*p* value
HPV self-sampling has the potential to help protect the health of my female patients.	147 (88)	133 (99)	<0.001
I feel informed about HPV self-sampling.	75 (45)	131 (97)	0.008
I am confident that I could talk with female patients about HPV self-sampling.	98 (59)	128 (95)	<0.001
	Mean score (SD)	Mean score (SD)	
On a scale from 1 to 10, with 1 being the lowest priority and 10 being the highest priority, how would you rate cervical cancer screening as a priority for your clinic?	8.3 (1.8)	8.6 (1.7)	<0.001

HPV, human papillomavirus; SD, standard deviation.

The postintervention survey also included six items that measured participants’ satisfaction with the intervention. Responses to these items were on a 5-point scale from strongly agree to strongly disagree. As with the attitude items described above, we dichotomized responses into either “agree” or “disagree.” These items included: the presentation was well-organized; the information in the presentation was easy to understand; I trust the information in the presentation; the presentation can help health care providers learn about HPV self-collection; based on the presentation, I am pleased that my clinic is taking part in the *HOME Initiative*; and the presentation took too long.

### Data analysis

We calculated descriptive statistics for demographic characteristics, knowledge, attitudes, and participant satisfaction. Change in the overall knowledge score from pre- to postintervention was determined using a linear mixed model with all available data and survey time point (pre- or postintervention survey) as the only covariate and a random effect for each participant.^34^ A similar approach using logistic mixed models was used to assess changes in each individual knowledge item and each attitude item. McNemar’s test for paired binary data (among only those participants with both pre- and postintervention survey data) was conducted as a sensitivity analysis. The results of McNemar’s test is included for only one survey item (HPV self-sampling has the potential to help protect the health of my female patients) where the logistic mixed model failed to converge. Results of other McNemar tests were similar to the mixed models and thus not reported. Data were analyzed using Stata Version 18 (College Station, Texas). All statistical tests were two-tailed with a critical alpha of 0.05.

## Results

### Participant characteristics

Participants had an average age of 42 years old. Most participants were females (92%), non-Hispanic white (95%), married/living together (74%), had at least a college degree (91%), and worked full-time (96%). Participants included physicians (15%), physician assistants (3%), nurse practitioners (36%), nurses (33%) or other staff (13%). On an average, physicians had worked at their health center for 6.1 years, while others had worked at their health center for an average of 4.8 years.

### Knowledge

The average number of correct responses to the seven knowledge items was 4.6 on the preintervention survey and 6.0 on the postintervention survey with an increase in the average number of correct responses of 1.4 (95% confidence interval [CI]: 1.2, 1.7; *p* < 0.001). There was an increase (*p* < 0.05) in the proportion of correct responses from preintervention to postintervention for all knowledge items except one (Table 2). The one item where an increase did not occur (“Women who have an HPV infection always have symptoms”) was answered correctly (*i.e.*, response of “false”) on the preintervention survey by nearly all participants (96%). Difference in improvement in knowledge by occupation categories was not statistically significant.

### Attitudes

The intervention significantly improved the attitudes of the participants about HPV self-collection and their confidence to talk to their female patients about HPV self-collection. Compared with the preintervention survey, participants were more likely to agree on the postintervention survey that HPV self-sampling had the potential to protect the health of patients (88% vs. 99%; *p* < 0.001), that they felt informed about HPV self-sampling (45% vs. 97%; *p* = 0.008), and that they were confident they could talk with patients about HPV self-sampling (59% vs. 95%; *p* < 0.001). Participants also reported that cervical cancer screening was a higher priority for their clinic on the postintervention survey compared with the preintervention survey (8.3 vs. 8.6; *p* < 0.001).

### Satisfaction with intervention

Participants reported high levels of satisfaction with the intervention. Nearly all participants agreed that the presentation was well-organized (99%), the information was easy to understand (99%), they trust the information (99%), and that the presentation can help health care providers learn about HPV self-sampling (99%). A large majority (97%) of participants also agreed that I am pleased that my clinic is taking part in the *HOME Initiative.* Only 15% of participants indicated agreement with the item: The presentation took too long.

## Discussion

HPV self-collection as a cervical cancer screening strategy is being used in some countries and is being examined in research studies in the United States to reach women who are not up-to-date with screening.^7,8,29^ In fact, in May 2024, the Food and Drug Administration approved expansion to the indications for women to self-collect a vaginal sample for HPV testing in a health care setting.^6^ Previously, we conducted a small pilot study associated with one health care system in Appalachian Ohio that demonstrated that a brief in-person educational intervention improved knowledge and attitudes about HPV self-collection among health care providers and staff.^22^ The current *HOME Initiative* educational intervention extends our previous work by providing information about HPV self-collection to health care providers and staff members practicing in Appalachian counties in four states where many women are not up-to-date with cervical cancer screening.

There were several significant findings from the provider-level educational intervention of the *HOME Initiative*. Results demonstrated improvement in knowledge and positive attitudes about cervical cancer and cervical cancer screening strategies, including HPV self-collection. In fact, there were specific knowledge items that had a low proportion answered correctly on the preintervention survey that improved following the intervention, thus helping to demonstrate that ongoing cancer screening education is important because of updates to cancer screening recommendations. In addition, following the intervention, there was an increase in providers feeling informed about HPV self-collection and reported an increase in their confidence to talk to patients about this screening strategy. This is a key finding since provider−patient communication will be key if HPV self-collection becomes a recommended screening strategy in the United States in the future. We think the positive effects of the intervention on knowledge and attitudes are especially promising given that the educational intervention is brief, and most providers reported high levels of satisfaction with the intervention.

The COVID-19 pandemic affected the health centers participating in this study and eliminated opportunities for in-person educational sessions. Faced with this dilemma, we revised the intervention for online delivery. This change in format for the educational sessions allowed providers and staff to complete the intervention online either individually or in a group setting. This change was critical because often in-person group delivery of an educational session is logistically difficult to arrange in a busy health care setting. In addition, by revising the intervention to be delivered online, it increases the potential scalability of this intervention for future dissemination efforts.

The study has several strengths. Participants were providers and staff members from health centers located in the Appalachian region of four states. The educational video was created by a professional media company to ensure technical quality. Also, as described above, the online format of the intervention provided flexibility to busy health care providers and staff. Limitations of the study include that some participants did not complete the postintervention survey. Reasons for this are unclear, but often a lack of time is an issue for busy health care providers and staff. This may have been especially true for educational sessions that were conducted during the peak of the COVID-19 pandemic. Participants were a convenience sample, and most were female and non-Hispanic white. However, these demographic characteristics reflect the characteristics of health care providers and staff practicing in this region of Appalachia.

Overall, this study showed that an online delivery of an educational cervical cancer screening intervention improved health care providers’ and staff members’ knowledge and attitudes about HPV self-collection. Previous studies have used in-person sessions to deliver educational interventions to increase cervical cancer screening.^25–28^ However, to our knowledge, the current study is one of the first to demonstrate the flexibility of an online provider educational intervention focused on cancer screening to be viewed individually or in a group setting in the United States. This is important since it may address several logistical issues associated with conducting in-person educational sessions in busy health centers.

## Conclusions

The use of home tests for a variety of medical conditions has increased in recent years, in part, due to the COVID-19 pandemic.^35^ Although HPV self-collection outside of a health care setting is not an approved or recommended screening approach in the United States, it is emerging as a potentially useful tool in the prevention of cervical cancer. Providing educational materials and programs to patients is important to increase cancer screening, however, it is also essential to provide educational materials and interventions to health care providers for the adoption of HPV self-collection if it becomes a recommended cervical cancer screening strategy in the United States in the future. The findings from this study suggest that a brief online educational intervention for health care providers and staff can improve knowledge and positive attitudes about cervical cancer prevention, including HPV self-collection. As importantly, participants reported high levels of satisfaction with the online educational session. Taken together, our findings suggest that our online educational session is a promising tool for educating health care providers and staff about HPV self-collection with the potential for wide dissemination.

## Abbreviations Used

CI: Confidence Interval
HOME Initiative: Health Outcomes through Motivation and Education
HPV: human papillomavirus
hrHPV: high-risk human papillomavirus
IRB: Institutional Review Board
Pap: Papanicolaou
RUCC: Rural-Urban Continuum Codes
SD: Standard Deviation
Take CARE Study: Clinical Avenues to Reach Health Equity
U.S.: United States
USPSTF: United States Preventive Services Task Force

## References

[1] Pollard K, Srygley SJ, Jacobsen LA. The Appalachian region: A data overview from the 2017-2021 American community survey. Chartbook. Appalachian Regional Commission, Population Reference Bureau 2023.

[2] Marshall JL, Thomas L, Lane NM, et al. Health disparities in Appalachia. 2017. Available from: arc.gov/report/health-disparities-in-Appalachia [Last accessed: June 10, 2024].

[3] Reiter PL, Fisher JL, Hudson AG, et al. Assessing the burden of HPV-related cancers in Appalachia. Hum Vaccin Immunother 2013;9(1):90–96; doi: 10.4161/hv.22389 23143774 PMC3667951

[4] Schoenberg NE, Studts CR, Hatcher-Keller J, et al. Patterns and determinants of breast and cervical cancer non-screening among Appalachian women. Women Health 2013;53(6):552–571; doi: 10.1080/03630242.2013.809400 23937729 PMC3812665

[5] Curry SJ, Krist AH, Owens DK, et al; US Preventive Services Task Force. Screening for cervical cancer: US preventive services task force recommendation statement. JAMA 2018;320(7):674–686; doi: 10.1001/jama.2018.10897 30140884

[6] U.S. Food & Drug Administration. FDA Roundup: May 17, 2024. Available from: https://www.fda.gov/news-events/press-announcements/fda-roundup-may-17-2024;2024 [Last accessed: July 10, 2024].

[7] Winer RL, Lin J, Tiro JA, et al. Effect of mailed human papillomavirus test kits vs usual care reminders on cervical cancer screening uptake, precancer detection, and treatment: A randomized clinical trial. JAMA Netw Open 2019;2(11):e1914729; doi: 10.1001/jamanetworkopen.2019.14729 31693128 PMC6865279

[8] Yeh PT, Kennedy CE, de Vuyst H, et al. Self-sampling for Human Papillomavirus (HPV) testing: A systematic review and meta-analysis. BMJ Glob Health 2019;4(3):e001351–e001351; doi: 10.1136/bmjgh-2018-001351 PMC6529022 31179035

[9] Montealegre JR, Anderson ML, Hilsenbeck SG, et al. Mailed self-sample HPV testing kits to improve cervical cancer screening in a safety net health system: Protocol for a hybrid effectiveness-implementation randomized controlled trial. Trials 2020;21(1):872–875; doi: 10.1186/s13063-020-04790-5 33087164 PMC7580009

[10] Ketelaars PJW, Bosgraaf RP, Siebers AG, et al. High-risk human papillomavirus detection in self-sampling compared to physician-taken smear in a responder population of the Dutch cervical screening: Results of the VERA study. Prev Med 2017;101:96–101; doi: 10.1016/j.ypmed.2017.05.021 28579497

[11] Tranberg M, Jensen JS, Bech BH, et al. Good concordance of HPV detection between cervico-vaginal self-samples and general practitioner-collected samples using the Cobas 4800 HPV DNA test. BMC Infect Dis 2018;18(1):348; doi: 10.1186/s12879-018-3254-y 30053836 PMC6062874

[12] Snijders PJF, Verhoef VMJ, Arbyn M, et al. High-risk HPV testing on self-sampled versus clinician-collected specimens: A review on the clinical accuracy and impact on population attendance in cervical cancer screening. Int J Cancer 2013;132(10):2223–2236; doi: 10.1002/ijc.27790 22907569

[13] Katz ML, Zimmermann BJ, Moore D, et al. Perspectives from health-care providers and women about completing Human Papillomavirus (HPV) self-testing at home. Women Health 2017;57(10):1161–1177; doi: 10.1080/03630242.2016.1243608 27700693 PMC5949215

[14] Bishop E, Katz ML, Reiter PL. Acceptability of human papillomavirus self-sampling among a national sample of women in the United States. Biores Open Access 2019;8(1):65–73; doi: 10.1089/biores.2018.0040 31057989 PMC6497327

[15] Nishimura H, Yeh PT, Oguntade H, et al. HPV self-sampling for cervical cancer screening: A systematic review of values and preferences. BMJ Glob Health 2021;6(5):e003743; doi: 10.1136/bmjgh-2020-003743 PMC8137189 34011537

[16] Mitchell EM, Lothamer H, Garcia C, et al. Acceptability and feasibility of community-based, lay navigator-facilitated at-home self-collection for human papillomavirus testing in underscreened women. J Womens Health (Larchmt) 2020;29(4):596–602; doi: 10.1089/jwh.2018.7575 31532298

[17] Katz ML, Reiter PL, Young GS, et al. Adherence to multiple cancer screening tests among women living in Appalachia Ohio. Cancer Epidemiol Biomarkers Prev 2015;24(10):1489–1494; doi: 10.1158/1055-9965.EPI-15-0369 26282630 PMC4592467

[18] Xiong S, Lazovich DA, Hassan F, et al. Health care personnel’s perspectives on Human Papillomavirus (HPV) self-sampling for cervical cancer screening: A pre-implementation, qualitative study. Implement Sci Commun 2022;3(1):130–133; doi: 10.1186/s43058-022-00382-3 36514133 PMC9745769

[19] Le A, Rohweder C, Wheeler SB, et al. Self-Collection for primary HPV testing: Perspectives on implementation from federally qualified health centers. Prev Chronic Dis 2023;20:E93; doi: 10.5888/pcd20.230056 37857461 PMC10599328

[20] Mao C, Kulasingam SL, Whitham HK, et al. Clinician and patient acceptability of self-collected human papillomavirus testing for cervical cancer screening. J Womens Health (Larchmt) 2017;26(6):609–615; doi: 10.1089/jwh.2016.5965 28332888 PMC5512311

[21] Pratt R, Winer R, Szpiro A, et al. Primary care provider and staff views on HPV self-sampling to address cervical cancer screening disparities. Annals of Family Medicine;21:3901; doi: 10.1370/afm.21.s1.3901

[22] Presser BE, Katz ML, Shoben AB, et al. Effects of an education intervention about HPV self-testing for healthcare providers and staff. J Cancer Educ 2018;33(5):954–959; doi: 10.1007/s13187-017-1164-0 28074444 PMC5503812

[23] U.S. Department of Agriculture. Economic Research Services. 2013 Rural-Urban Continuum Codes. U.S. Department of Agriculture; 2024.

[24] Appalachian Regional Commission. Classifying economic distress in appalachian counties. County Economic Status and Distressed Areas by State. Appalachian Regional Commission: 2024.

[25] Curry WJ, Lengerich EJ, Kluhsman BC, et al. Academic detailing to increase colorectal cancer screening by primary care practices in Appalachian Pennsylvania. BMC Health Serv Res 2011;11:112–112; doi: 10.1186/1472-6963-11-112 21600059 PMC3128846

[26] Dignan M, Shelton B, Slone SA, et al. Effectiveness of a primary care practice intervention for increasing colorectal cancer screening in Appalachian Kentucky. Prev Med 2014;58:70–74; doi: 10.1016/j.ypmed.2013.10.018 24212061 PMC3925970

[27] Mader EM, Fox CH, Epling JW, et al. A practice facilitation and academic detailing intervention can improve cancer screening rates in primary care safety net clinics. J Am Board Fam Med 2016;29(5):533–542; doi: 10.3122/jabfm.2016.05.160109 27613786 PMC13054612

[28] Gorin SS, Ashford AR, Lantigua R, et al. Effectiveness of academic detailing on breast cancer screening among primary care physicians in an underserved community. J Am Board Fam Med 2006;19(2):110–121; doi: 10.3122/jabfm.19.2.110 16513899

[29] Reiter PL, Shoben AB, McDonough D, et al. Results of a pilot study of a mail-based human papillomavirus self-testing program for underscreened women from Appalachian Ohio. Sex Transm Dis 2019;46(3):185–190; doi: 10.1097/OLQ.0000000000000944 30461597 PMC6370501

[30] Serrano B, Brotons M, Bosch FX, et al. Epidemiology and burden of HPV-related disease. Best Pract Res Clin Obstet Gynaecol 2018;47:14–26; doi: 10.1016/j.bpobgyn.2017.08.006 29037457

[31] Hariri S, Unger ER, Sternberg M, et al. Prevalence of genital human papillomavirus among females in the United States, the national health and nutrition examination survey, 2003-2006. J Infect Dis 2011;204(4):566–573; doi: 10.1093/infdis/jir341 21791659

[32] Fisher JL, Englehardt HL, Stephens JA, et al. Cancer-related disparities among residents of Appalachia Ohio. J Health Dispa Res Pra 2008;2:4.

[33] Hall HI, Uhler RJ, Coughlin SS, et al. Breast and cervical cancer screening among Appalachian women. Cancer Epidemiol Biomarkers Prev 2002;11(1):137–142. 11815412

[34] Xi W, Pennell ML, Andridge RR, et al. Comparison of intent-to-treat analysis strategies for pre-post studies with loss to follow-up. Contemp Clin Trials Commun 2018;11:20–29; doi: 10.1016/j.conctc.2018.05.008 30023456 PMC6022256

[35] Kullgren J, Solway E, Singer D, et al. National Poll on Healthy Aging: At-Home Medical Tests. University of Michigan 2022.

